# A chicken liver cell line efficiently supports the replication of ALV-J possibly through its high level viral receptor and efficient protein expression system

**DOI:** 10.1186/s13567-018-0537-7

**Published:** 2018-05-02

**Authors:** Tuofan Li, Jing Xie, Lu Lv, Shu Sun, Xiaomei Dong, Quan Xie, Guangcheng Liang, Chichao Xia, Hongxia Shao, Aijian Qin, Jianqiang Ye

**Affiliations:** 1 Key Laboratory of Jiangsu Preventive Veterinary Medicine, Key Laboratory for Avian Preventive Medicine, Ministry of Education, College of Veterinary Medicine, Yangzhou University, Yangzhou, 225009 Jiangsu China; 2 Jiangsu Co-innovation Center for Prevention and Control of Important Animal Infectious Diseases and Zoonoses, Yangzhou, 225009 Jiangsu China; 3 Joint International Research Laboratory of Agriculture and Agri-Product Safety, The Ministry of Education of China, Yangzhou University, Yangzhou, 225009 Jiangsu China; 4 Institute of Agricultural Science and Technology Development, Yangzhou University, Yangzhou, 225009 Jiangsu China

## Abstract

In this study, we identified a chicken liver cell line (LMH) which could strongly support the replication of ALV-J (Subgroup J of avian leukosis virus) with high viral titer. Notably, ALV-J was efficiently detected by ELISA in LMH cells 1 day before DF1 cells. In comparison with DF1 cells, LMH cells not only expressed higher levels of ALV-J receptor chNHE-1, but also possessed a more efficient protein expression system for foreign genes. Thus, LMH cells could be a novel tool to shorten the ALV-J eradication approach and accelerate studies on the pathogenesis and oncogenesis of ALV-J.

## Introduction, methods and results

Avian leukosis viruses (ALV) can be divided into seven subgroups (A–E, J and K) in the chicken based on the antigenic pattern of the envelope protein. Unlike other subgroups, ALV-J infection mainly induces hematopoietic malignancy with myeloid leukemia and hemangioma [1–3]. Since its first report in 1988 from the UK, ALV-J has spread globally and caused great economic losses in the poultry industry. Due to the lack of a vaccine and anti-viral drugs, the eradication program is the only efficient way to control ALV-J so far [3]. In the ALV-J eradication approach, viral isolation in DF1 cells is the gold standard for ALV-J detection. Traditionally, clinical samples (such as blood, cloaca swab, meconium and sperm) are inoculated into DF1 cells and cultured for 7–9 days before detection by ELISA or IFA, which is time-consuming and less efficient [4–6]. Therefore, shortening the time line for ALV-J isolation is critical for efficient ALV-J eradication. A chicken liver cell line (LMH) was used to explore whether other cell lines could replace DF1 cells for the replication of ALV-J.

**Figure 1:**
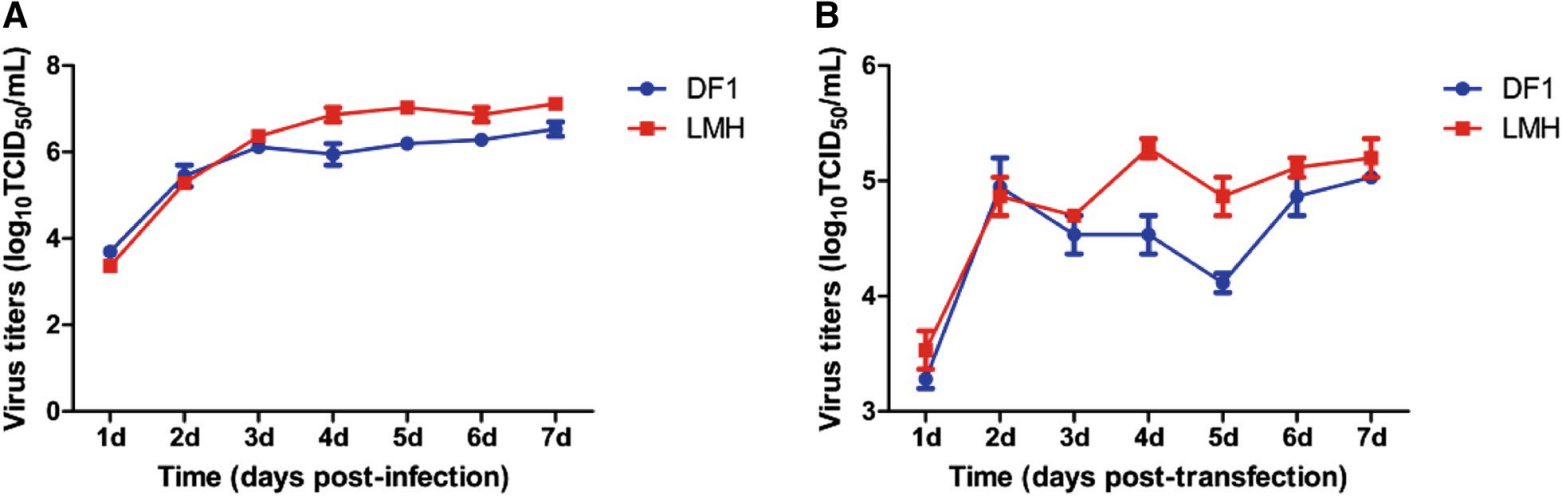
**The viral replication kinetics of ALV-J in DF1 and LMH cells. A** Viral growth curve of ALV-J. DF1 and LMH cells were infected with ALV-J GY03 at MOI 0.001 respectively, and the supernatant of the infected cells were collected at the indicated time points and titrated by TCID_50_; **B** comparison of viral rescuing efficiency in DF1 and LMH cells. DF1 and LMH cells were transfected with 4 μg of ALV-J infectious clone respectively and the supernatant of the transfected cells were collected at the indicated time points and titrated by TCID_50_.

The growth curve of ALV-J in two kinds of cells was generated in order to compare the replication ability of ALV-J in DF1 and LMH cells. In brief, DF1 and LMH cells with 70% confluence in the 6-well plate were infected with ALV-J at an MOI of 0.001 respectively. The supernatant (200 μL) from the infected cells was collected at the indicated time points and titrated in DF1 cells by IFA. The TCID_50_ of these supernatants were determined by the Reed-Muench method and the viral growth curves were constructed by GraphPad Prism 5 software. As described in Figure 1, although ALV-J showed similar viral titers in both cell lines at 1, 2 and 3 dpi (day post-infection), the virus at 4, 5, 6 and 7 dpi showed significant higher titer in LMH cells than that in DF1 cells. The peak titer of ALV-J in LMH cells reached 1.58 × 10^7^ TCID_50_/mL whereas that in DF1 cells was 5 × 10^6^ TCID_50_/mL. It should be noted that the viral titer in LMH cells at 4 dpi reached 1.08 × 10^7^ TCID_50_/mL which was higher than the peak titer in DF1 cells at 7 dpi. To compare the efficacy of viral rescuing in the two kinds of cell lines, the ALV-J infectious clone was transfected into DF1 and LMH cells respectively. Briefly, 4 μg plasmid was first mixed evenly with 200 μL Opti-MEM, then the transfection reagent MIRUS (8 μL) was added and mixed softly. The transfection mixture was incubated at room temperature for 45 min and then added into the fresh DF1 and LMH cells in 6-well plates respectively. The transfection mixture was replaced with fresh medium containing 1% FBS 6 h post-transfection. The viruses in the supernatant from the cells transfected with ALV-J infectious clone were collected at the indicated time points and were titrated in DF1 cells. During the ALV-J rescuing study in both cell lines, the similar viral replication kinetics was found as shown in Figure 1B. Again, the rescued ALV-J in LMH cells at 4 dpi had a higher titer than that in DF1 cells at 7 dpi. These data clearly demonstrate that LMH cells support the replication of ALV-J more efficiently than DF1 cells.

**Figure 2:**
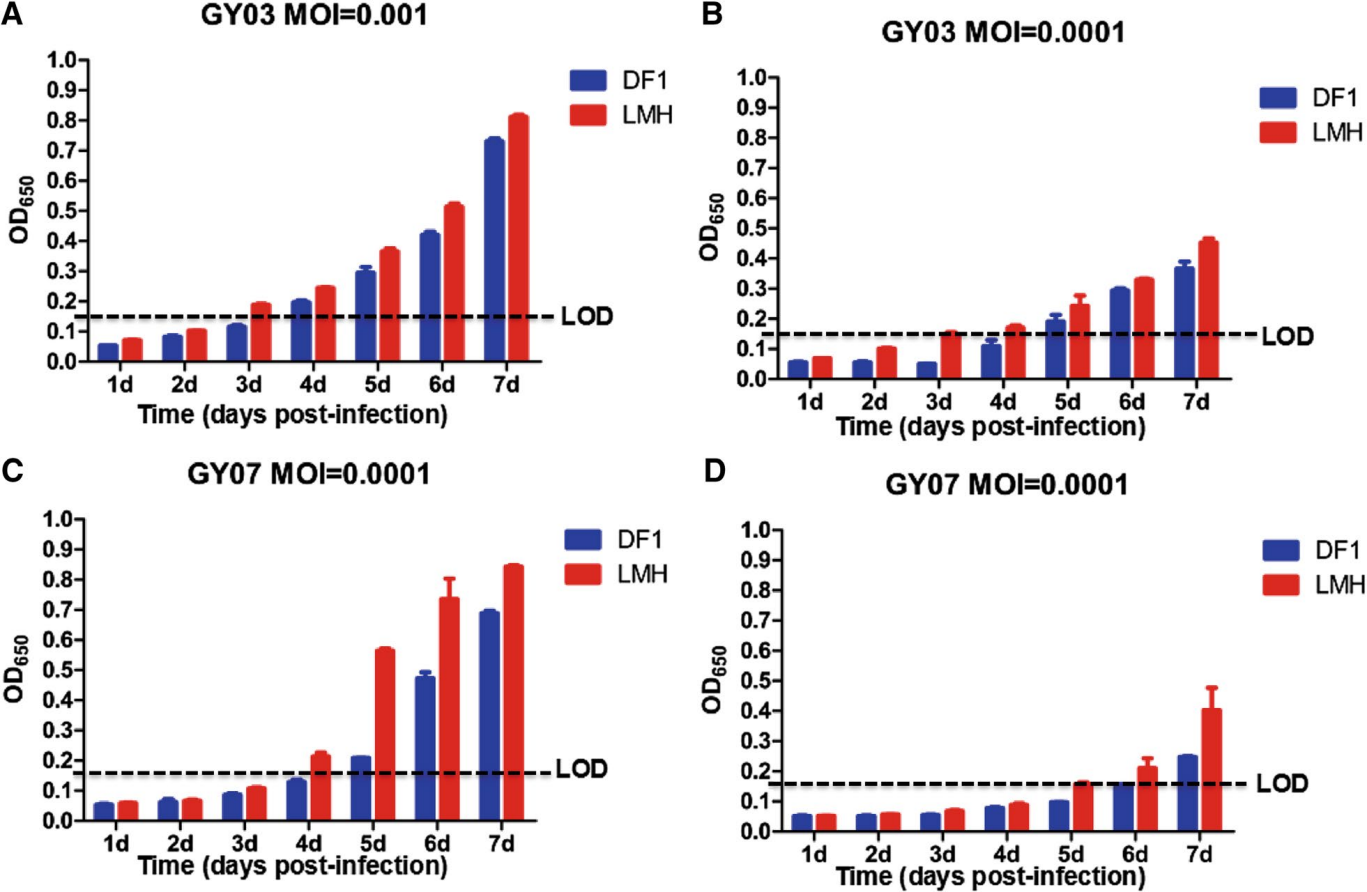
**Comparison of the ALV-J detection with ELISA between DF1 and LMH cells.** DF1 and LMH cells were infected with ALV-J GY03 and GY07 at MOI 0.001 (**A**, **C**) or MOI 0.0001 (**B**, **D**) respectively, and the supernatant of the infected cells were collected at the indicated time points and the p27 antigen in the supernatant was detected by ELISA.

**Figure 3:**
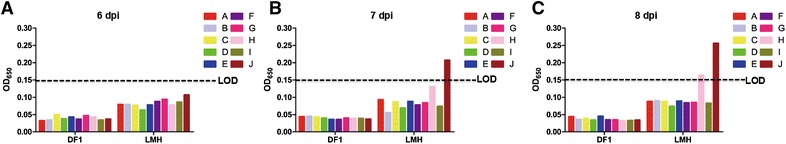
**Comparison of clinical sample detection with ELISA between DF1 and LMH cells.** DF1 and LMH cells were inoculated with homogenates of chicken liver and spleen samples, respectively, and the supernatant of the infected cells were collected at 6 dpi (**A**), 7 dpi (**B**) or 8 dpi (**C**). The p27 antigen in the supernatant was detected by ELISA.

To evaluate whether LMH cells could replace DF1 cells to shorten the time line for ALV-J isolation and identification during an eradication program, both cell lines were infected with ALV-J GY03 at MOI of 0.001 and 0.0001 respectively, and the supernatant from the infected cells was collected at different time points for ELISA detection. In the ELISA, mAb 5D3 was coated into ELISA plates to capture the p27 antigen, and mAb 4F12-HRP was used to detect the captured antigen [7, 8]. The OD_650_ value was determined after adding TMB as a substrate and 1% SDS to stop the reaction. An OD_650_ value of 0.15 was the cut-off of the ELISA. As shown in Figures 2A and B, the p27 antigen of ALV-J was detected in LMH cells earlier for 1 and 2 days than in DF1 cells when the infection dose was 0.001 and 0.0001 MOI respectively. To confirm this data, the viral growth kinetics of another ALV-J isolate GY07 was tested in both cell lines. As described in Figures 2C and D, the p27 antigen of ALV-J was also detected in LMH cells earlier for 1 day than in DF1 cells when the infection dose was 0.001 and 0.0001 MOI respectively. To further evaluate whether the LMH cells were suitable for detection of ALV in clinical samples, 10 homogenates of liver or spleen from chickens with susceptible infection of ALV were inoculated into both DF1 and LMH cells respectively. The supernatant from the inoculated cells were collected at different time points and detected by ELISA. As described in Figure 3, ELISA results for two samples J and H from LMH cells were positive at days 7 and 8 post-inoculation respectively, whereas ELISA results for the samples from DF1 cells were negative (Figure 3). To confirm the two positive samples, a blind passage of the two samples in LMH cells was performed. The OD_650_ value of the two positive samples in ELISA increased to 0.921 and 0.904 respectively at day 5 post-inoculation. These data clearly demonstrate that LMH cells were more sensitive than DF1 cells for detection of ALV in clinical samples. These results highlight that LMH can be used as a novel cell line for ALV-J isolation and identification to shorten the ALV-J eradication approach.

**Figure 4:**
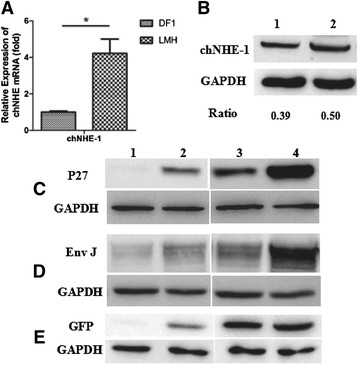
**Expression level of ALV-J receptor chNHE-1 and protein expression level for foreign genes in DF1 and LMH cells. A** Transcription level of chNHE-1 in DF1 and LMH cells were detected with real-time PCR. **B** Western blot analysis for the expression of chNHE-1 in the DF1 and LMH cells. Lane 1, DF1 cells; lane 2, LMH cells. The gray ratio values of chNHE-1 to GAPDH are shown. **C**–**E** Western blot analysis for DF1 cells and LMH cells transfected with 1 μg pc-ALV-p27, pc-ALV-J-env and pc-eGFP respectively, at 12 h and 24 h. Lane 1 and 2, DF1 and LMH cells transfected with the plasmids indicated for 12 h, respectively; Lane 3 and 4, DF1 and LMH cells transfected with the indicated plasmids for 24 h, respectively.

chNHE-1 (Na+/H+ exchanger type 1) is the first identified viral receptor for ALV-J [9]. In order to investigate the molecular mechanisms involved in LMH cell support of ALV-J replication, the expression level of ALV-J receptor chNHE-1 was detected both in mRNA and protein level using qRT-PCR and Western blot respectively. Real-time PCR was performed as previously described [10]. The sequences of primers for chNHE-1 are the following: F: GAGGAGGAAGAGGAAGAAGATG; R: CTGAACAAACAGCACCACAAC. In the Western blot, DF1 and LMH cells were lysed with lysis buffer (CST) on ice for 30 min. Then, the lysates were analyzed by Western blot as previously described [10]. As described in Figure 4A, the mRNA level of chNHE-1 in LMH cells was significantly higher than that in DF1 cells (*P* value < 0.05). This data was further confirmed by the analysis of protein levels of chNHE-1 in both LMH and DF1 cells as described in Figure 4B. The high level of ALV-J receptor chNHE-1 in LMH cells may contribute to the efficient replication of ALV-J in LMH cells.

In addition to high levels of ALV-J receptors expressed in LMH, the protein expression level for foreign genes in the transfected LMH cell were also investigated. pc-ALV-p27, pc-ALV-J-env and pc-eGFP plasmids were transfected into DF1 and LMH cells respectively and the transfected cells were lysed at the indicated time point. Western blot was performed as described above. As shown in Figures 4C and D, the expression level of ALV-J viral p27 and Env proteins in the transfected LMH cells were much higher than those in the transfected DF1 cells at 12 hpt (hour post-transfection). Notably, the plasmid pcDNA3.1-eGFP in LMH cells also had significantly higher expression levels of eGFP than that in DF1 cells at 12 hpt (Figure 4E). Although the expression level of these proteins in both transfected cells were similar at 24 hpt, only very weak bands were found for DF1 cells at 12 hpt whereas clear and strong bands appear for LMH cells. These data demonstrate that the protein expression system in LMH cells is much more efficient than that in DF1 cells.

## Discussion

It is well known that avian leukosis is one of the vital serious tumor diseases in poultry. In addition to different kinds of tumors, the infection of ALV generally results in immunosuppression in chickens that significantly affects the sustained development of the poultry industry globally [11]. Since no vaccine and anti-viral drug against ALV is now available, the eradication program has been strictly applied in developed countries to control the diseases caused by ALV. The emerging of novel ALV subgroups (such as ALV-J and ALV-K) challenges the current ALV eradication approaches [3, 12–14]. Although many methods have been developed for detection of ALV-J during the stamping-out program, the isolation of ALV-J using DF1 cells is still a well-established standard approach for ALV-J detection. However, the time and low efficacy of the current ALV-J isolation in DF1 cells does not meet well with the requirement for massive detection of ALV-J for its eradication in developing countries, which requires more efficient viral isolation and detection systems for ALV-J. In this study, a chicken liver cell line, LMH, was found to efficiently support the viral replication of ALV-J. Compared to DF1 cells, ALV-J in LMH not only replicated faster, but also yielded higher viral titers. This efficient viral replication of ALV-J in LMH cells can shorten the time of ELISA detection during ALV-J isolation and identification as described in Figure 2. Notably, although DF1 cells were free of the endogenous ALV-E whereas the gp85 mRNA of ALV-E was detected in the LMH cells by RT-PCR (data not shown), both Western blot and ELISA did not detect the endogenous p27 protein in LMH cells. The clinical detection also showed that LMH cells were more sensitive than DF1 cells for detection of ALV. These results indicate that DF1 cells could be replaced by LMH cells for ALV-J detection.

Since the viral receptor in the cells is critical to ALV infection and replication, the finding of the high level of expression of ALV-J receptor chNHE-1 in the LMH cell can explain why ALV-J replicates efficiently and yields higher viral titers in LMH cells when compared with DF1 cells. It should be mentioned that we also detected another two newly identified cell receptors for ALV-J (GRP78 and ANXA2) in both DF1 and LMH cells [10, 15]. However, there was no significant difference for these receptors between the two cell lines (data not shown). The high expression level of chNHE-1 in LMH cells was consistent with the previous study on the distribution of chNHE-1 in the primary chicken liver cell, indicating that the chicken liver cell might be efficient ALV-J target cells [16]. However, whether ALV-J could transform chicken liver cells needs to be further investigated. Because the metabolism of the liver cells are thought to be more active than that of other kinds of cells, we also compared the expression level of foreign genes in both DF1 and LMH cells through transfection studies. Interestingly, we found that both ALV-J related plasmids and ALV-J non-related plasmids could be expressed early in LMH cells with higher levels than that in DF1 cells. The early expression of foreign genes or viral genes in LMH cells with high levels might also contribute to the efficient replication of ALV-J in LMH cells and to the shortening of the time line for ALV-J detection through the good standard method by using LMH cells.

In conclusion, this is the first demonstration that a chicken liver cell line, LMH, with a high level of ALV-J receptors and efficient protein expression system could efficiently support the replication of ALV-J, and could be used as a novel tool to rapidly isolate and identify ALV-J, and in accelerating the ALV-J eradication program and studies on the pathogenesis and oncogenesis of ALV-J. It should also be noted that the characteristics of high-level expression for foreign genes in LMH cells indicates that the LMH cells might also improve viral replication for other viruses and be used to efficiently rescue viruses, if the LMH cell carries the corresponding receptor.
